# Susceptibility and severity of COVID-19 and risk of psychiatric disorders in European populations: a Mendelian randomization study

**DOI:** 10.3389/fpsyt.2023.1253051

**Published:** 2023-10-05

**Authors:** Hua Xue, Li Zeng, Shuangjuan Liu

**Affiliations:** ^1^Department of Neurology, Sichuan Taikang Hospital, Chengdu, China; ^2^Department of Respiratory, Affiliated Hospital of Youjiang Medical University for Nationalities, Baise, China; ^3^Department of Neurology, Qionglai People’s Hospital, Chengdu, China

**Keywords:** COVID-19, SARS-CoV-2, psychiatric disorders, schizophrenia, bipolar disorder, anxiety disorders

## Abstract

**Background:**

Observational studies have suggested that COVID-19 increases the prevalence of psychiatric disorders, but the results of such studies are inconsistent. This study aims to investigate the association between COVID-19 and the risk of psychiatric disorders using Mendelian randomization (MR) analysis.

**Methods:**

We used summary statistics from COVID-19 Host Genetics Initiative genome-wide association study (GWAS) of COVID-19 involving 2,586,691 participants from European ancestry. Genetic variations of five psychiatric disorders including autism spectrum disorder (ASD) (*N* = 46,351), bipolar disorder (BID) (*N* = 51,710), major depressive disorder (MDD) (*N* = 480,359), anxiety disorder (*N* = 83,566), and schizophrenia (SCZ) (*N* = 77,096) were extracted from several GWAS of European ancestry. The inverse-variance weighted (IVW) method as the main MR analysis conducted. We further performed sensitivity analyzes and heterogeneity analyzes as validation of primary MR results.

**Results:**

The IVW analysis found that COVID-19 hospitalization phenotype was the risk factor for BID (OR = 1.320, 95% CI = 1.106–1.576, *p* = 0.002) and SCZ (OR = 1.096, 95% CI = 1.031–1.164, *p* = 0.002). Moreover, we detected a significant positive genetic correlation between COVID-19 severity and two psychiatric traits, BID (OR = 1.139, 95% CI = 1.033–1.256, *p* = 0.008) and SCZ (OR = 1.043, 95% CI = 1.005–1.082, *p* = 0.024). There was no evidence supporting the causal relationship between COVID-19 susceptibility and psychiatric disorders.

**Conclusion:**

Our results found that the COVID-19 hospitalization phenotype and COVID-19 severity phenotype might be the potential risks of BID and SCZ in European populations. Therefore, patients infected with SARS-CoV-2 should have enhanced monitoring of their mental status.

## 1. Introduction

Coronavirus disease 2019 (COVID-19) is caused by the severe acute respiratory syndrome coronavirus 2 (SARS-CoV-2) infection, which usually has respiratory symptoms as the main clinical manifestation and can also lead to multisystem involvement (1–3). Since the COVID-19 outbreak in December 2019, the World Health Organization has classified the COVID-19 outbreak as an international public health emergency (4, 5). Some clinical investigations suggest that the COVID-19 epidemic is a highly stressful event, which acts as an important stressor disrupting the physiological and psychological balance of individuals. This imbalance can potentially lead to various degrees of mental health problems in both social groups and individuals (6). Among patients in COVID-19 epidemic areas, the more common mental symptoms are nervousness, anxiety, worry, fear, insomnia and other symptoms or various symptoms of physical discomfort. In severe cases, some patients even develop psychiatric disorders, such as major depressive disorder and anxiety disorder, or relapse of their original mental illness (7–9). However, the strength and significance of the observed associations of COVID-19 with psychiatric disorders remain controversial.

An observational study involving 56,679 participants conducted in China showed that the risk of depression was 3.27 [95% confidence interval (CI): 1.84–5.80], anxiety disorder was 2.48 (95% CI: 1.43–4.31), and insomnia was 3.06 (95% CI: 1.73–5.43) in patients with COVID-19 (10). A cohort study conducted in the United States comprising 153,848 people showed that the risk of psychiatric disorders in patients with COVID-19 was 1.46 (95% CI: 1.40–1.52), including 1.41 (95% CI: 1.40–1.52) for sleep disorders, 1.38 (95% CI: 1.34–1.43) for stress disorders, and 1.35 (95% CI: 1.30–1.39) for anxiety disorders (11). A cross-sectional population study in South Korea showed that participants with moderate or severe depressive symptoms and anxiety symptoms accounted for 12.6 and 6.8%, respectively (12). In addition, during the COVID-19 pandemic, the disruption of the daily lives, physical activities, social life and educational progress of children and adolescents also had an impact on their physical and mental health, with symptoms such as anxiety, depression, post-traumatic stress disorder (PTSD), sleep problems and non-suicidal self-injury (13). A cross-sectional survey conducted after 2 months of COVID-19 quarantine in Saudi Arabia revealed that among adolescents, 15.5% had no symptoms, 44.1% experienced mild symptoms, and 13.0% exhibited potential PTSD symptoms (14).

SARS-CoV-2 infection or the presence of residual virus may lead to persistent psychiatric symptoms such as brain fog, memory loss, and decreased thinking and reaction ability (15). Additionally, the immune response triggered by the virus can have a long-lasting impact on the brain and other organs, including hormone feedback systems and blood biochemical transduction signaling systems (16). These effects can potentially trigger various biological responses. For example, under chronic stress, the brain signals the adrenal gland to release cortisol for extended durations, resulting in a malfunctioning hormone system and an overactive immune system. These factors contribute to an increased susceptibility to anxiety, depression, and other mental disorders (17).

A growing number of observational studies suggest that COVID-19 may potentially trigger the onset of mental disorders, such as anxiety disorders, depression, and even schizophrenia. However, traditional observational studies are often interfered by a variety of confounding factors, such as living environment, education level, and eating habits. Mendelian randomization (MR) is a causal inference method that relies on genetic variation. Its fundamental principle is to utilize the impact of randomly assigned genotypes on phenotypes in nature to infer the influence of biological factors on diseases, which can largely avoid potential confounding factors and reverse causality (18). Therefore, MR analysis, as an epidemiological method, is widely used to verify the causal relationship found in observational studies (19). In short, MR studies use genetic variation as an instrumental variables (IVs) to avoid confounding factors and reverse causality (20). In this study, we conducted a two-sample MR study to assess the causal relationship between COVID-19 and five important psychiatric disorders included autism spectrum disorder (ASD), major depressive disorder (MDD), bipolar disorder (BID), schizophrenia (SCZ), and anxiety disorder as outcomes.

## 2. Methods

### 2.1. Study design

We conducted a two-sample MR analysis to investigate the causal relation of COVID-19 traits on the risk of psychiatric traits (21). It is well known that MR studies are carried out under the assumption that instrumental variable (IVs) associated with exposure is independent of known or unknown confounders and that IVs affects outcomes only through exposure and not through other pathways (19). In this MR study, three COVID-19 traits (COVID-19 susceptibility, COVID-19 hospitalization, COVID-19 severity) as exposure, five psychiatric traits including autism spectrum disorder (ASD), major depressive disorder (MDD), bipolar disorder (BID), schizophrenia (SCZ), and anxiety disorder as outcomes.

### 2.2. Data sources and genetic instruments

We obtained summary-level data for COVID-19 susceptibility, hospitalization, severity from the latest version of the COVID-19 Host Genetics Initiative (HGI) GWAS meta-analyzes, round 6 (22). Diagnosis of COVID-19 cases relies on laboratory-confirmed infection of SARS-CoV-2, as well as electronic health record documentation or physician diagnosis of COVID-19. Additionally, self-reported COVID-19 infection from the patient is also considered. The exposure of COVID-19 susceptibility phenotype compared 112,612 European COVID-19 patients with a control population of 2,474,079 without a history of COVID-19. Patients who were diagnosed with COVID-19 and hospitalized due to COVID-19 were considered as a COVID-19 hospitalized cohort. The exposure of COVID-19 hospitalization phenotype compared 24,274 patients who were hospitalized due to COVID-19 with a control group (*N* = 2,061,529) consisting of individuals who were diagnosed with COVID-19 but were not hospitalized or were free of COVID-19. The COVID-19 severe cohort includes hospitalized patients who died from COVID-19, and those who developed respiratory failure and needed respiratory support (including tracheotomy, tracheal intubation, non-invasive ventilator-assisted ventilation, invasive ventilator-assisted ventilation, etc.) The exposure of COVID-19 severity phenotype compared 8,779 severe hospitalized individuals with a controls who were without severe COVID-19, or who were free of COVID-19 (*N* = 1,001,875).

We obtained summary-level data for five psychiatric traits from published multiplied studies with large sample sizes of European ancestry (23). Genome-wide association study data for Autism Spectrum Disorder (ASD), Major Depressive Disorder (MDD), Bipolar Disorder (BID), and Schizophrenia (SCZ) were sourced from the Psychiatric Genomics Consortium (PGC) (24–27). The PGC is an international consortium of scientists committed to meta-analysis of genome-wide genetic data, specifically focusing on psychiatric disorders. Summary-level data for anxiety disorders were obtained from a meta-analyzed, involving 83,566 participants (25,453 cases and 58,113 controls) from the UK Biobank (28, 29). The basic characteristics of GWASs, including exposures and outcomes, are listed in Table 1.

**Table 1:** Detailed information of the studies and datasets used for Mendelian randomization analyzes.

Traits	Population	Sample size (cases/controls)	Data source	PMID
Exposure				
COVID-19 susceptibility	Europeans	112,612/2,474,079	COVID-19 HGI	32,404,885
COVID-19 hospitalization	Europeans	24,274/2,061,529	COVID-19 HGI	32,404,885
COVID-19 severity	Europeans	8,779/1,001,875	COVID-19 HGI	32,404,885
Outcome				
ASD	Europeans	18,382/27,969	PGC	33,686,288
MDD	Europeans	135,458/344,901	PGC	30,718,901
BID	Europeans	20,352/31,358	PGC	34,002,096
SCZ	Europeans	33,640/43,456	PGC	35,396,580
Anxiety disorder	Europeans	25,453/58,113	UKB	31,748,690

ASD, autism spectrum disorder; MDD, major depressive disorder; BID, bipolar disorder; SCZ, Schizophrenia; UKB, the UK Biobank; PGC, the Psychiatric Genomics Consortium; COVID-19 HGI, COVID-19 Host Genetics Initiative.

### 2.3. IVs selection

**Figure 1:**
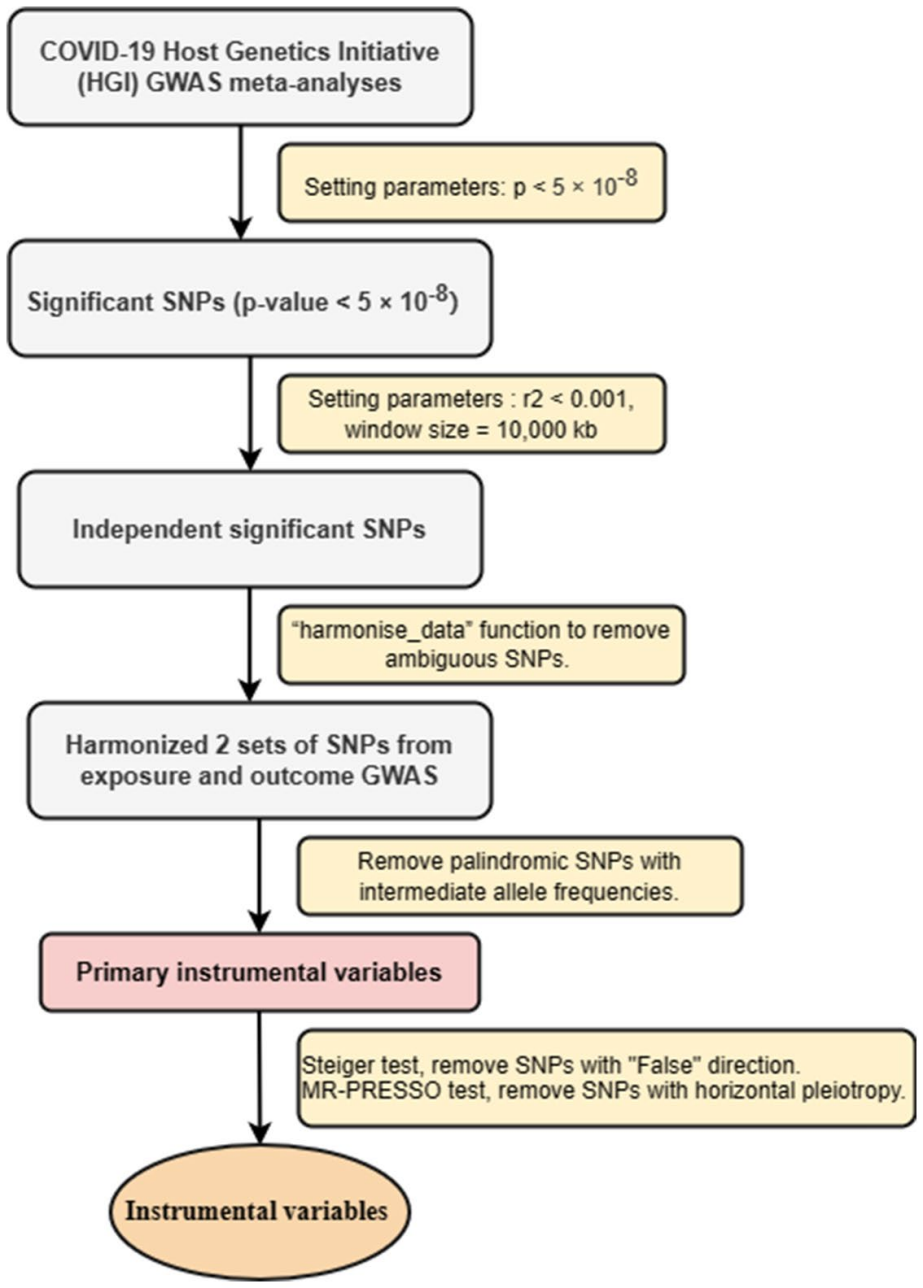
Flowchart of instrumental variables selection strategy.

To ensure that all screened IVs meet MR analysis standards, we have adopted a series of strict control steps. First step, to ensure the relevance of IVs, we extracted genome-wide significant single nucleotide polymorphisms (SNPs) from exposed GWASs. Only SNPs that value of *p* < 5 × 10^−8^ were considered strongly associated with exposure used as IVs. In the second step, to ensure the independence of IVs, we conducted linkage disequilibrium (LD) clumping (*r*^2^ < 0.001, window size = 10,000 kb) to select independent significant SNPs. In the third step, using the screened SNPs, we extracted SNPs from the outcome (psychiatric disorders) GWAS (30). We harmonized the exposure and outcome datasets using the “harmonise_data” function to remove ambiguous SNPs and create a new data frame combining exposure and outcome data. For SNPs not found in the outcome GWAS, after reconciling the above two sets of SNPs, palindromic SNPs with intermediate allele frequencies were removed, and the remaining SNPs were retained as primary IVs. In the fourth step, we conducted Steiger test, removed SNPs with “False” direction. We perfomed MR-PRESSO test, removed SNPs with horizontal pleiotropy (29). At same time, we used the Pheno Scanner database to examine selected IVs associated with other phenotypes that might influence the results (31). The detailed information for the selected SNP is shown in the Supplementary File. IVs screening flow chart is shown in Figure 1.

### 2.4. Statistical analysis

To address the potential pleiotropic effects of genetic variation, this study applied three MR analyzes to assess the causal effects of COVID-19 traits on psychiatric disorders. We applied the standard inverse variance weighting (IVW) method as the primary MR methods, which combined the Wald ratio of each SNPs on the outcome and obtained a pooled causal estimate. If there is heterogeneity, we use IVW random effect method. In addition, MR-Egger and weighted median (WM) methods, as further complementary methods to MR, these methods can provide more reliable estimates in a broader range of situations (31). MR-Egger regression can provide tests for unbalanced pleiotropy and considerable heterogeneity, whereas for the same underexposed variation it requires a larger sample size (20). MR-Egger method often yields inaccurate and statistically less significant results, especially when the number of SNPs is small. In addition, the value of the MR-Egger intercept term was far from zero, indicating horizontal pleiotropy (*p* < 0.05) (32, 33). Therefore, in our MR study, the MR-Egger method was mainly performed to detect pleiotropy. The WM method will return an unbiased estimate if more than one-half of the IVs were valid.

Horizontal pleiotropy occurs when exposure (COVID-19) related genetic variations directly affect the results by assuming multiple pathways other than exposure (psychiatric disorders). Therefore, we further conducted Cochrane’ s Q statistic, leave-one-out (LOO) analysis and MR-Egger intercept test to detect the existence of pleiotropy and evaluate the robustness of the results (33). When the *p* value of the Cochrane Q test is less than 0.05, there is heterogeneity. We also evaluate the horizontal multidirectionality based on the intercept term obtained by MR-Egger regression (34). In order to determine whether the causal estimation is driven by a single SNP, we performed a LOO analysis, in which each exposure-related SNP was discarded in turn to repeat the IVW analysis.

## 3. Results

### 3.1. Association of COVID-19 susceptibility with psychiatric traits

**Figure 2:**
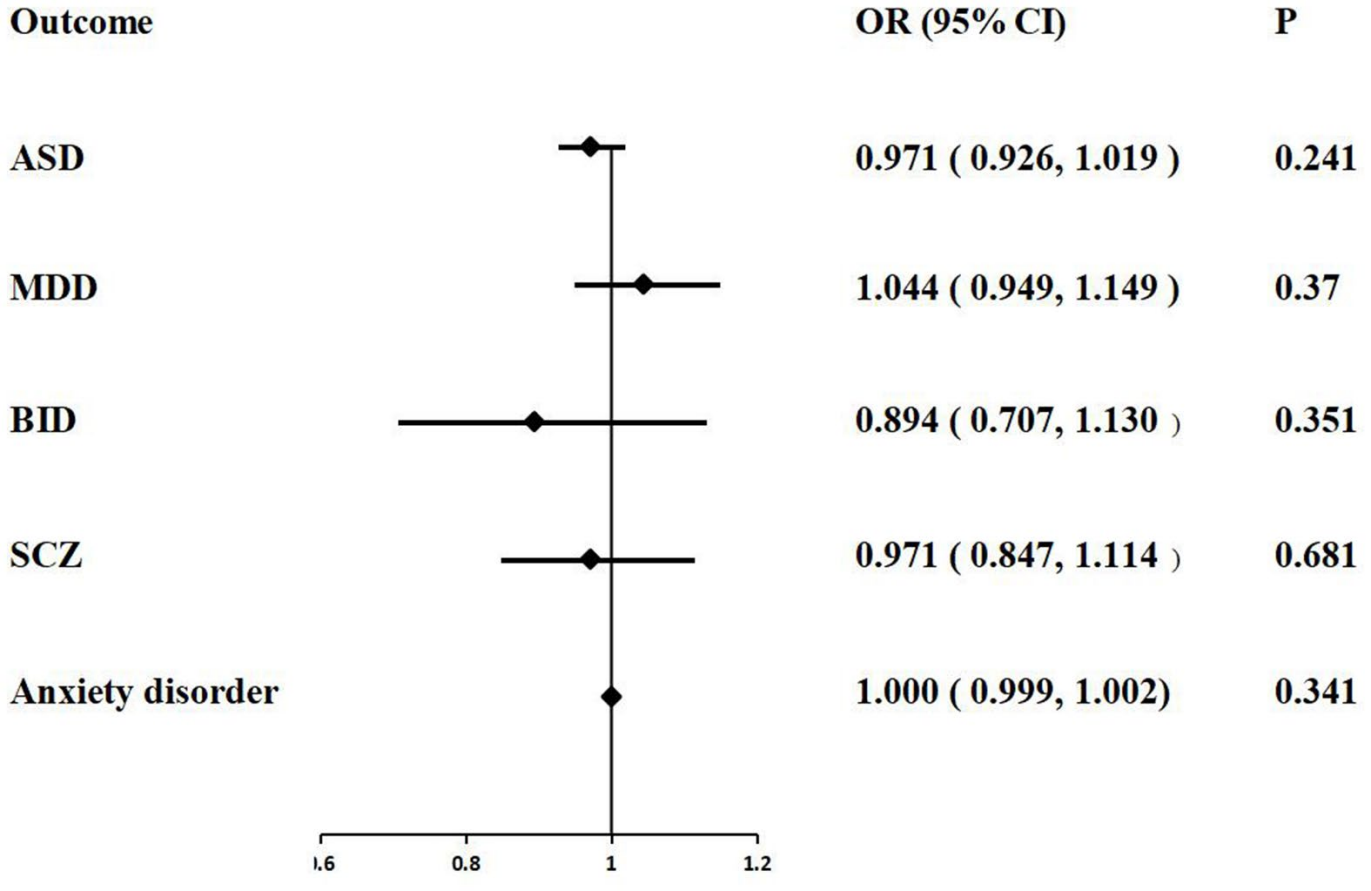
Associations of COVID-19 susceptibility with five psychiatric traits based on the IVW method. ASD, autism spectrum disorder; MDD, major depressive disorder; BID, bipolar disorder; SCZ, Schizophrenia.

The IVW MR analysis suggested that there is no evidence supporting COVID-19 susceptibility as a risk or protective factor for five psychiatric traits, ASD (IVW: OR = 0.971, 95% CI = 0.926–1.019, *p* = 0.241), MDD (IVW: OR = 1.044, 95% CI = 0.949–1.149, *p* = 0.370), BID (IVW: OR = 0.894, 95% CI = 0.707–1.130, *p* = 0.351), SCZ (IVW: OR = 0.971, 95% CI = 0.847–1.114, *p* = 0.681), anxiety disorder (IVW: OR = 1.000, 95% CI = 0.999–1.002, *p* = 0.341). The MR results are shown in Table 2 and Figure 2.

**Table 2:** MR estimates for the causal effect of COVID-19 susceptibility on psychiatric disorder.

Exposure	Outcome	IVW		Weighted median		MR-Egger		Cochran Q test		MR-Egger	
		OR (95% CI)	*p*	OR (95% CI)	*p*	OR (95% CI)	*p*	Q value	*p*	Intercept	*p*
COVID-19 susceptibility	ASD	0.971 (0.926, 1.019)	0.241	1.013 (0.978, 1.493)	0.461	1.078 (0.985, 1.618)	0.241	1.077	0.583	−0.008	0.229
COVID-19 susceptibility	MDD	1.044 (0.949, 1.149)	0.370	1.019 (0.896, 1.158)	0.769	1.278 (0.920, 1.776)	0.239	3.913	0.417	−0.019	0.296
COVID-19 susceptibility	BID	0.894 (0.707, 1.130)	0.351	0.840 (0.643, 1.005)	0.056	0.998 (0.396, 2.464)	0.981	8.571	0.072	−0.009	0.836
COVID-19 susceptibility	SCZ	0.971 (0.847, 1.114)	0.681	0.942 (0.816, 1.088)	0.420	1.285 (0.859, 1.923)	0.308	7.472	0.112	−0.028	0.248
COVID-19 susceptibility	Anxiety disorder	1.001 (0.999, 1.002)	0.341	1.005 (0.988, 1.021)	0.351	1.000 (0.998, 1.292)	0.484	4.958	0.291	0.002	0.329

The Cochrane’ s Q test suggested that there was no heterogeneity in the main MR analysis among the five psychiatric traits (all *p* values >0.05). Additionally, no horizontal pleiotropy was found, with an insignificant intercept from the MR-Egger test (all *p* values >0.05). The results of leave-one-out sensitivity analyzes suggested that the causal associations between COVID-19 susceptibility traits and psychiatric disorders were not affected by any individual SNP (Supplementary Figures S1–S5).

### 3.2. Association of COVID-19 hospitalization with psychiatric traits

**Figure 3:**
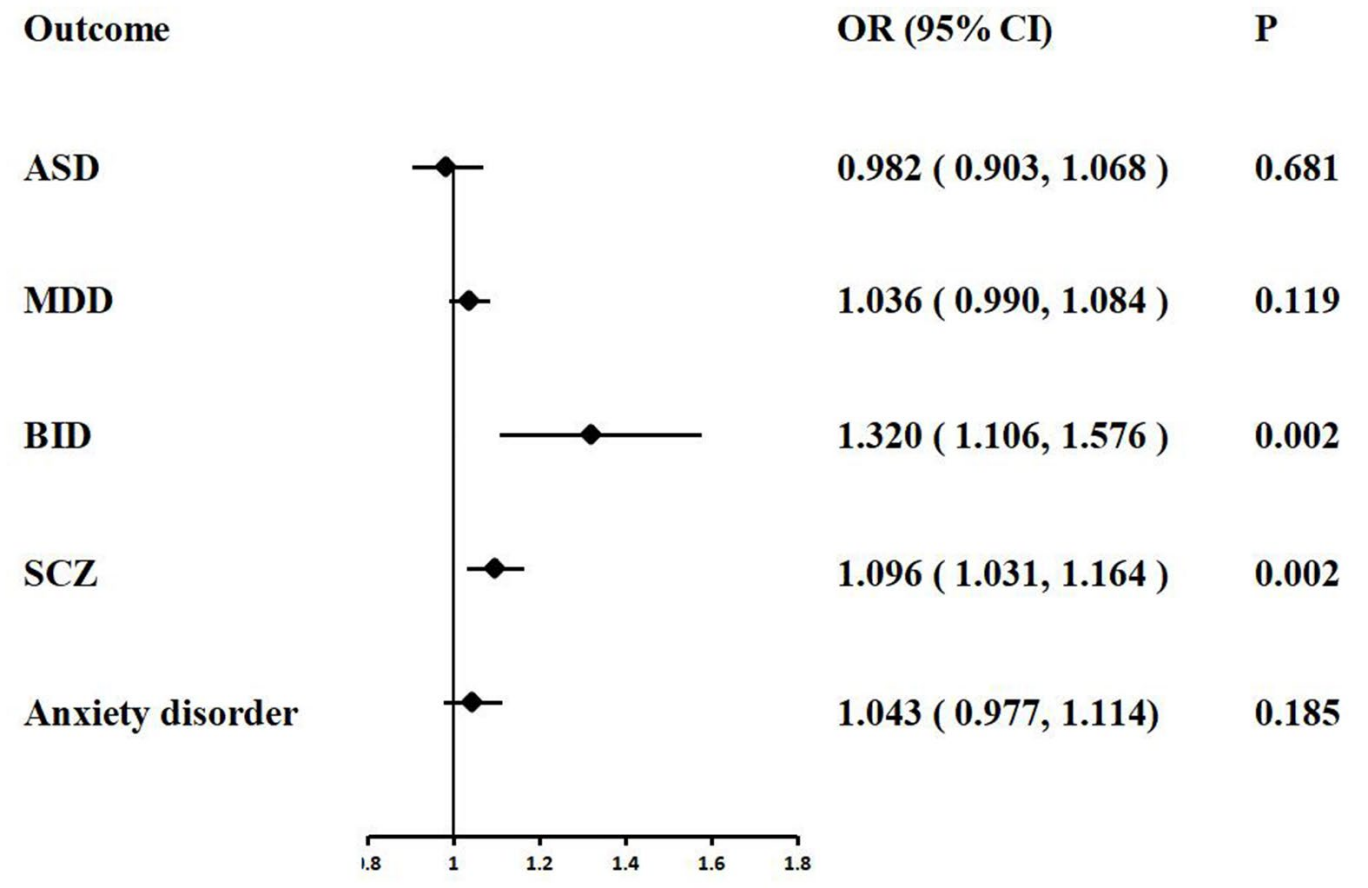
Associations of COVID-19 hospitalization with five psychiatric traits based on the IVW method. ASD, autism spectrum disorder; MDD, major depressive disorder; BID, bipolar disorder; SCZ, Schizophrenia.

In the IVW analyzes, one unit increase in log odds of hospitalization of COVID-19 was suggestively associated with higher BID risk (IVW: OR = 1.320, 95% CI = 1.106–1.576, *p* = 0.002) and SCZ risk (IVW: OR = 1.096, 95% CI = 1.031–1.164, p = 0.002). The IVW MR analysis suggested that there is no evidence supporting COVID-19 hospitalization trait as a risk or protective factor for ASD (IVW: OR = 0.982, 95% CI = 0.903–1.068, *p* = 0.681), MDD (IVW: OR = 1.036, 95% CI = 0.990–1.084, *p* = 0.119), and anxiety disorder (IVW: OR = 1.043, 95% CI = 0.977–1.114, *p* = 0.185). The MR results are shown in Table 3 and Figure 3.

**Table 3:** MR estimates for the causal effect of COVID-19 hospitalization on psychiatric disorder.

Exposure	Outcome	IVW		Weighted median		MR-Egger		Cochran Q test		MR-Egger	
		OR (95% CI)	*p*	OR (95% CI)	*p*	OR (95% CI)	*p*	Q value	*p*	Intercept	*p*
COVID-19 hospitalization	ASD	0.982 (0.903, 1.068)	0.681	0.967 (0.869, 1.075)	0.537	1.609 (0.702, 3.690)	0.323	5.717	0.334	−0.090	0.306
COVID-19 hospitalization	MDD	1.036 (0.990, 1.084)	0.119	1.034 (0.979, 1.093)	0.223	0.990 (0.626, 1.564)	0.968	1.359	0.928	0.008	0.853
COVID-19 hospitalization	BID	1.320 (1.106, 1.576)	**0.002**	1.345 (1.086, 1.665)	**0.006**	1.493 (0.737, 3.027)	0.327	6.102	0.296	0.139	0.338
COVID-19 hospitalization	SCZ	1.096 (1.031, 1.164)	**0.002**	1.069 (0.990, 1.155)	0.085	1.540 (0.839, 2.829)	0.235	4.832	0.436	−0.062	0.331
COVID-19 hospitalization	Anxiety disorder	1.043 (0.977, 1.114)	0.185	1.035 (0.955, 1.123)	0.392	1.066 (0.518, 2.194)	0.869	0.553	0.990	−0.003	0.957

Bold values suggest that there are significant differences in statistics.

Weak evidence of directional pleiotropy was found in the MR Egger intercept tests (all *p* values >0.05). The Cochrane’ s Q test suggested that there was no heterogeneity in the main MR analysis among the five psychiatric traits (all p values >0.05). The results of leave-one-out sensitivity analyzes suggested that the causal associations between COVID-19 susceptibility traits and psychiatric disorders were not affected by any individual SNP (Supplementary Figures S5–S10).

### 3.3. Association of COVID-19 severity with psychiatric traits

**Figure 4:**
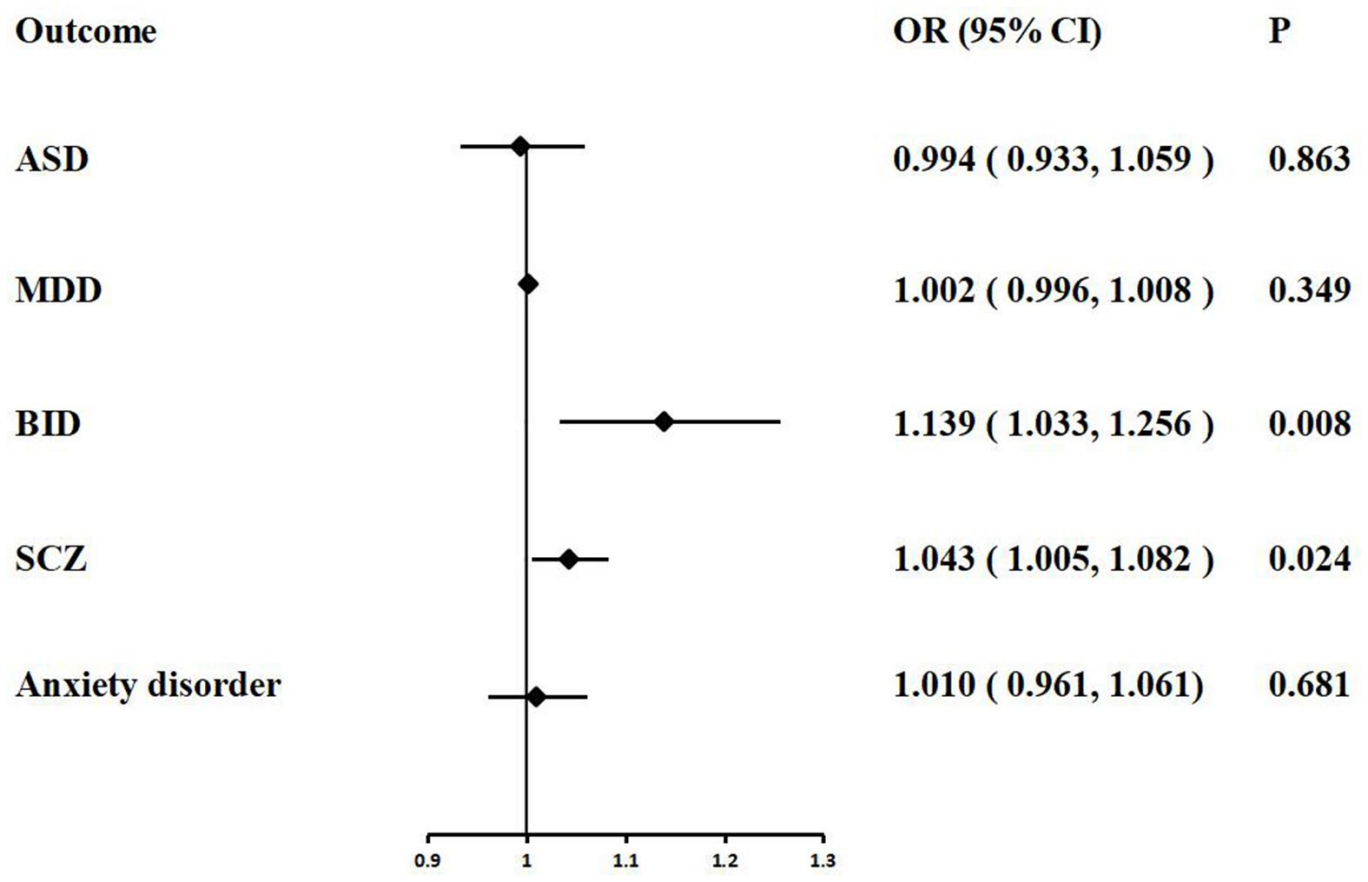
Associations of COVID-19 severity with five psychiatric traits based on the IVW method. ASD, autism spectrum disorder; MDD, major depressive disorder; BID, bipolar disorder; SCZ, Schizophrenia.

In the MR analysis, we detected a significant positive genetic correlation between COVID-19 severity and two psychiatric traits, BID (IVW: OR = 1.139, 95% CI = 1.033–1.256, *p* = 0.008) and SCZ (IVW: OR = 1.043, 95% CI = 1.005–1.082, *p* = 0.024). The IVW MR analysis suggested that there is no evidence supporting COVID-19 susceptibility as a risk or protective factor for ASD (IVW: OR = 0.994, 95% CI = 0.933–1.059, *p* = 0.863), MDD (IVW: OR = 1.002, 95% CI = 0.996–1.008, *p* = 0.349), and anxiety disorder (IVW: OR = 1.010, 95% CI = 0.961–1.061, *p* = 0.681). The MR results are shown in Table 4 and Figure 4.

**Table 4:** MR estimates for the causal effect of COVID-19 severity on psychiatric disorder.

Exposure	Outcome	IVW		Weighted Median		MR-Egger		Cochran Q test		MR-Egger	
		OR (95% CI)	*p*	OR (95% CI)	*p*	OR (95% CI)	*p*	Q value	*p*	Intercept	*p*
COVID-19 severity	ASD	0.994 (0.933, 1.059)	0.863	0.975 (0.892, 1.066)	0.586	1.070 (0.784, 1.461)	0.689	4.963	0.420	−0.018	0.658
COVID-19 severity	MDD	1.002 (0.996, 1.008)	0.349	1.004 (0.996, 1.011)	0.279	1.004 (0.975, 1.035)	0.777	4.976	0.418	−0.004	0.906
COVID-19 severity	BID	1.139 (1.033, 1.256)	**0.008**	1.145 (1.005, 1.304)	**0.040**	0.792 (0.500, 1.255)	0.367	4.198	0.649	0.088	0.174
COVID-19 severity	SCZ	1.043 (1.005, 1.082)	**0.024**	1.047 (0.996, 1.102)	0.069	1.092 (0.914, 1.304)	0.374	5.521	0.478	−0.011	0.627
COVID-19 severity	Anxiety disorder	1.010 (0.961, 1.061)	0.681	1.025 (0.964, 1.090)	0.418	0.959 (0.775, 1.186)	0.719	5.174	0.394	0.013	0.645

Bold values suggest that there are significant differences in statistics.

We performed extensive sensitivity analyzes to validate the association between COVID-19 severity and the risk of psychiatric disorders. The Cochran’ s Q test did not detect the heterogeneity of effects across the IVs (all *p* values >0.05, Table 4). No apparent horizontal pleiotropy was observed as the intercept of MR-Egger was not significantly deviated from zero (Table 4). The leave-one-out results suggest that the causal effect was not driven by a single instrumental variable (Supplementary Figures S11–S15).

## 4. Discussion

In this study, we investigated the association of three COVID-19 traits (COVID-19 susceptibility, COVID-19 hospitalization, and COVID-19 severity) on five psychiatric traits (ASD, MDD, BID, SCZ, and anxiety disorder) using MR analysis for the first time. A risk effect was found in COVID-19 hospitalization and COVID-19 severity. Specifically, hospitalization of COVID-19 increased the risk of BID and SCZ. Moreover, COVID-19 severity also increased the risk of BID and SCZ. There is no evidence supporting COVID-19 susceptibility as a risk or protective factor for five psychiatric traits.

Our findings on the increased genetic susceptibility risk for BID and SCZ due to COVID-19 hospitalization and COVID-19 severity. Our study does not support the genetic susceptibility of COVID-19 and anxiety disorder which aligns with recent epidemiological observations in the United States and the United Kingdom (9, 35). The incidence of mental health symptoms and psychiatric disorders is higher in COVID-19 patients. One reason may be that the SARS-CoV-2 increases the risk of psychiatric disorders in patients by increasing the levels of inflammatory factors, such as interleukin-6 (IL-6) and interleukin-8 (IL-8), in the blood and cerebrospinal fluid (36). In a cohort study, the risk of new psychiatric disorders was 2.87 times (95% CI: 2.45–3.35) higher in patients with severe COVID-19 than in those with mild infections (37). This may be because critically ill patients are prone to cerebral hypoxia, which increases the occurrence of psychiatric symptoms through mechanisms such as neuronal dysfunction, brain edema, and increased blood–brain barrier permeability (38). In addition to SARS-CoV-2 infection, the main epidemic factors affecting public mental health also include isolation and unemployment. A survey from Hong Kong showed that the unemployment rate in Hong Kong increased from 3.7 to 4.2% during the COVID-19 pandemic (39). Unemployment usually has a negative psychological impact on individuals, making them prone to anxiety and depression. A prospective longitudinal study in the United Kingdom found that public anxiety and depression increased in the early stages of isolation and improved as the isolation measures were gradually relaxed (40). A retrospective study from China found an increased risk of first-onset schizophrenia in older adults at the beginning of the COVID-19 outbreak compared with a similar period from 2017 to 2019 (41).

Neuropsychiatric symptoms are a significant aspect of the long-term effects of COVID-19. These symptoms commonly include cognitive impairment, sleep disturbances, depression, anxiety, post-traumatic stress symptoms, and substance use disorders (42). A meta-analysis of mid and long-term neurological and neuropsychiatric manifestations of post-COVID-19 syndrome, which included 1,458 articles, showed a significant increase in the prevalence of neuropsychiatric disorders including sleep disorders, anxiety, and depression (42). However, the mechanisms underlying COVID-19 neuropsychiatric symptoms are still poorly understood. In the acute phase of COVID-19, research data indicate that the pathophysiological basis of neuropsychiatric injury mainly includes hypoxemia, hyperinflammatory state, and hypercoagulable state (43). The increase of pro-inflammatory cytokines, especially IL-6, is a characteristic of moderate to severe COVID-19, which can cause endothelial dysfunction, increase vascular permeability, and aggravate blood–brain barrier (BBB) dysfunction (44). The neuropathological data of the COVID-19 death patients suggested endothelial injury, microbleeds, microvascular basal layer destruction, and fibrinogen extravasation into the brain parenchyma (43). The above pathological changes suggested that the BBB was ruptured, which may be mediated by the new coronary-related inflammatory state. At the same time, strong inflammation in turn leads to a hypercoagulable state, and further causes microthrombus formation and microvascular endothelial damage (45). Cumulative static brain injury, hypoxia, inflammation, BBB dysfunction, and autoimmune are the mechanisms of psychiatric disorders in the late stage of COVID-19 (46). The persistence of autoimmunity and the presence of the virus can lead to chronic inflammation in patients with COVID-19. This chronic inflammation, especially characterized by imbalances in IL-6 cytokine levels, has been found to be associated with anxiety, depression, and traumatic stress (46). It is well known that the SARS-CoV-2 virus enters the brain by mediating the angiotensin-converting enzyme-2 (ACE-2) receptor and has a great impact on the central nervous system (47). Various inflammatory mediators, such as cytokines, chemokines, and various metabolites, are poorly regulated during infection, as well as in several psychiatric disorders, leading to brain tissue hypoxia and cytokine storm syndrome. Persistence of SARS-CoV-2 infection may also lead to exacerbation of pre-existing neuropsychiatric symptoms in patients (47). Yet the SARS-CoV-2 infection during the COVID-19 epidemic was not the only factor contributing to the occurrence of psychiatric disorders. A systematic review of neuropsychological and psychiatric sequalae of COVID-19 suggested that factors that emerging risk factors for psychiatric symptoms include female sex, perceived stigma related to COVID-19, infection of a family member, social isolation, and prior psychiatry history (48).

The advantages of using MR analysis design in this study are as follows. First, we use randomly assigned genetic variants to identify the causal effects of exposure (three important COVID-19 traits) on the results (five psychiatric traits). Based on the three basic assumptions of MR, we can reduce conventional bias and avoid reverse causality. Second, the SNPs strongly associated with COVID-19 and psychiatric traits selected in this study are from GWASs with a large sample size, which increases the reliability when interpreting the causal effect of the results. Third, this study selected five psychiatric traits, including anxiety disorders, autism spectrum disorder, major depressive disorder, bipolar disorder, and schizophrenia, in order to fully illustrate the causal relationship between COVID-19 and psychiatric disorders. Fourth, the conclusion became more convincing by confirming our results with several methods (IVW, WM, and MR-egger), and sensitivity tests (Cochran’ Q test, LOO analysis, and MR-Egger intercept test).

There are several limitations in this study that need to be acknowledged. Firstly, for three COVID-19 traits GWAS summary statistics used in this MR study were not stratified by age of onset and gender. Previous observational studies have shown that elderly patients are more likely to suffer from schizophrenia in the early stage of COVID-19 outbreak. Thus, MR analyzes could not be performed to assess the causal effect of different COVID-19 populations on psychiatric traits. Secondly, although the population of this study is from European descent. However, there may be differences in the diagnosis and treatment of COVID-19 in different European country regions, and there may be significant cross-regional differences in the prevalence as well as predictors of psychiatric disorders. Therefore, the conclusions of this study need to be viewed with caution, and cannot be applied to populations in regions such as Asia and Africa. Thirdly, despite a series of rigorous measures to screen for IVs, however, we are still unable to completely rule out SNPs with pleiotropic effect, and it is difficult to discuss the extent to which the conclusions are influenced by this phenomenon. Fourthly, epigenetic issues such as DNA methylation, RNA editing and transposon inactivation are inevitable pitfalls of MR analysis.

## 5. Conclusion

In conclusion, this MR study provided suggestive genetic evidence for the associations of COVID-19 hospitalization traits and COVID-19 severity traits with increased risks of BID and SCZ. Therefore, patients infected with SARS-CoV-2 should have enhanced monitoring of their psychiatric status. Further studies are required to illuminate the effectiveness of timely treating COVID-19 on reducing the risk of psychiatric disorder and investigate the potential mechanisms of these association.

## Data availability statement

The original contributions presented in the study are included in the article/supplementary material. Further inquiries can be directed to the corresponding authors.

## Author contributions

HX and LZ carried out the conception and design of the research. HX participated in the acquisition of data and carried out the analysis and interpretation of data. HX and SL drafted the manuscript. SL participated in the revision of the manuscript for important intellectual content. All authors contributed to the article and approved the submitted version.
