# Supplementary material for: Susceptibility and severity of COVID-19 and risk of psychiatric disorders in European populations: a Mendelian randomization study
Source: Front Psychiatry. 2023 Oct 5;14:1253051. doi: 10.3389/fpsyt.2023.1253051 (PMC10585067; doi:10.3389/fpsyt.2023.1253051)
Supplement: Supplementary file 1 [file Data_Sheet_1.docx]

Susceptibility and severity of COVID-19 and risk of psychiatric disorders in European populations: A Mendelian randomization study

Hua Xue^1*^, Li Zeng^2^, Shuangjuan Liu^3^

^1^Department of Neurology, Sichuan Taikang Hospital, Chengdu, Sichuan, 610213, China

^2^Department of Respiratory, Affiliated Hospital of Youjiang Medical University for Nationalities, Baise, Guangxi, 533000, China

^3^Department of Neurology, Qionglai People’ s Hospital, Chengdu, Sichuan, 610213, China

*** Correspondence:**Corresponding Author : Hua Xue
E-mail : [xueh1895@163.com](mailto:xueh1895@163.com)

***Supplementary materials***

| **Instrumental variables of COVID-19 susceptibility** | | | | | |
| --- | --- | --- | --- | --- | --- |
| SNP | Effect_allele | Other_allele | EAF | SE | P value |
| rs4971066 | G | T | 0.1777 | 0.0134 | 1.02E-08 |
| rs10936744 | T | C | 0.3588 | 0.0099836 | 3.51E-10 |
| rs17078348 | G | A | 0.0997 | 0.016154 | 1.20E-08 |
| rs643434 | A | G | 0.371 | 0.010114 | 1.29E-23 |
| rs757405 | A | T | 0.7092 | 0.010783 | 1.64E-10 |
| rs12482060 | G | C | 0.3375 | 0.010525 | 3.96E-09 |
| **Instrumental variables of COVID-19 hospitalization** | | | | | |
| SNP | Effect_allele | Other_allele | EAF | SE | P value |
| rs41264915 | G | A | 0.08167 | 0.035776 | 1.02E-08 |
| rs35081325 | T | A | 0.08587 | 0.035359 | 7.93E-54 |
| rs111837807 | C | T | 0.1004 | 0.034274 | 3.22E-10 |
| rs10860891 | A | C | 0.8547 | 0.033124 | 3.43E-08 |
| rs1859330 | A | G | 0.6979 | 0.022603 | 4.91E-12 |
| rs2109069 | A | G | 0.322 | 0.023578 | 1.96E-15 |
| rs13050728 | C | T | 0.6382 | 0.023893 | 6.72E-15 |
| **Instrumental variables of COVID-19 severity** | | | | | |
| SNP | Effect_allele | Other_allele | EAF | SE | P value |
| rs35081325 | T | A | 0.07529 | 0.044502 | 5.75E-45 |
| rs111837807 | C | T | 0.0996 | 0.04276 | 5.66E-12 |
| rs2237698 | T | C | 0.08971 | 0.039653 | 2.41E-09 |
| rs2384074 | T | C | 0.6756 | 0.02821 | 2.10E-12 |
| rs10860891 | A | C | 0.8855 | 0.039713 | 1.64E-09 |
| rs77534576 | T | C | 0.03465 | 0.074941 | 8.52E-10 |
| rs2109069 | A | G | 0.3287 | 0.02807 | 6.12E-20 |
| rs13050728 | C | T | 0.6627 | 0.028559 | 2.44E-12 |

**
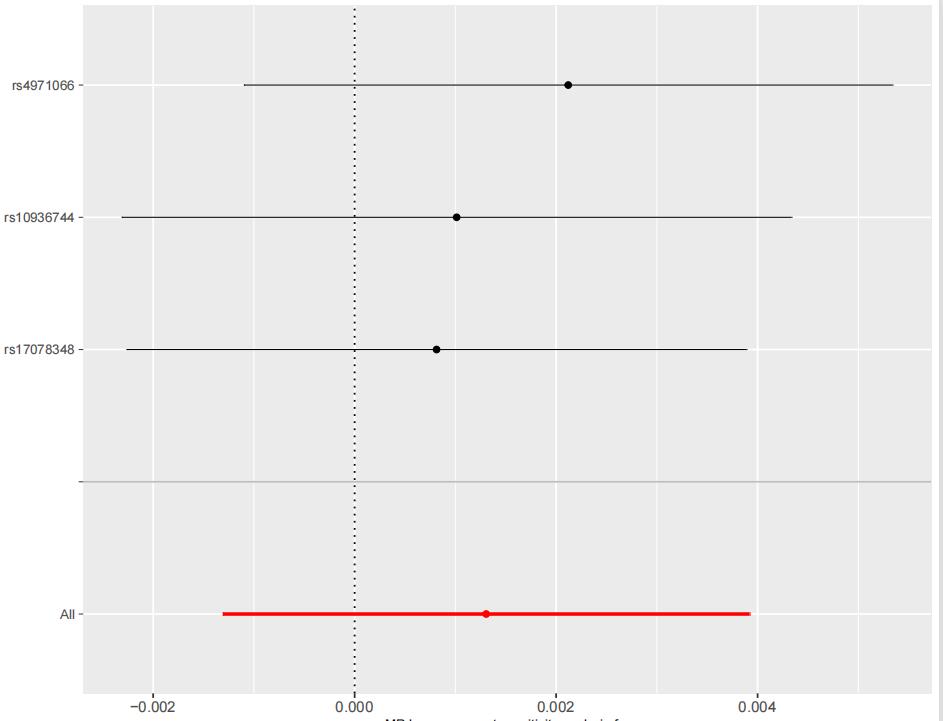
**

**Figure S1** Leave-one-out sensitivity analyses for COVID-19 susceptibility on anxiety disorders.


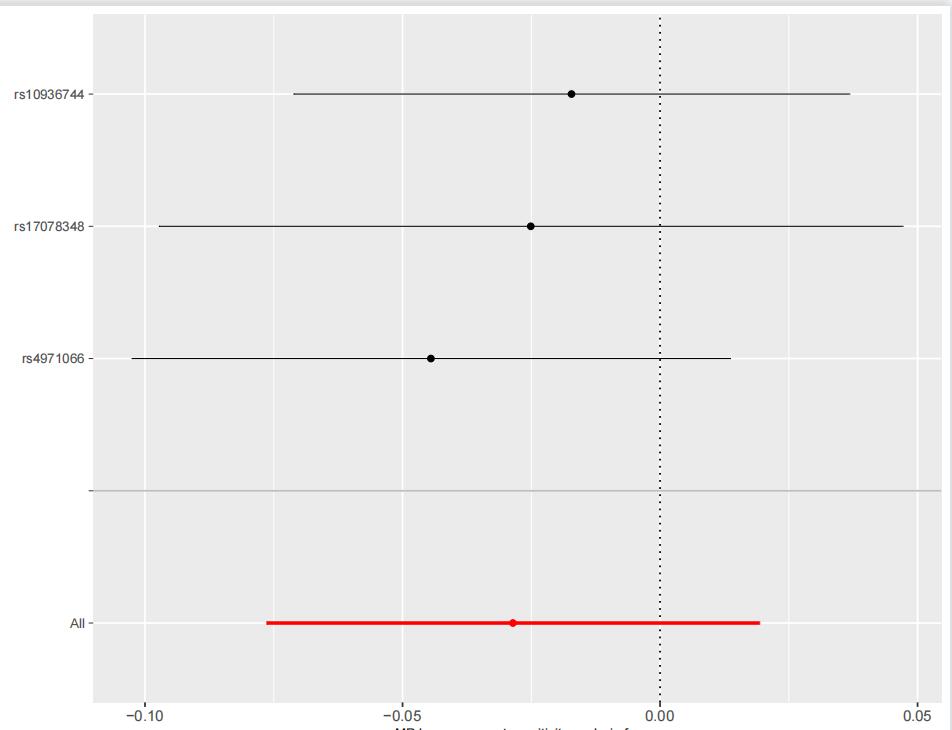


**Figure S2** Leave-one-out sensitivity analyses for COVID-19 susceptibility on autism spectrum disorder.


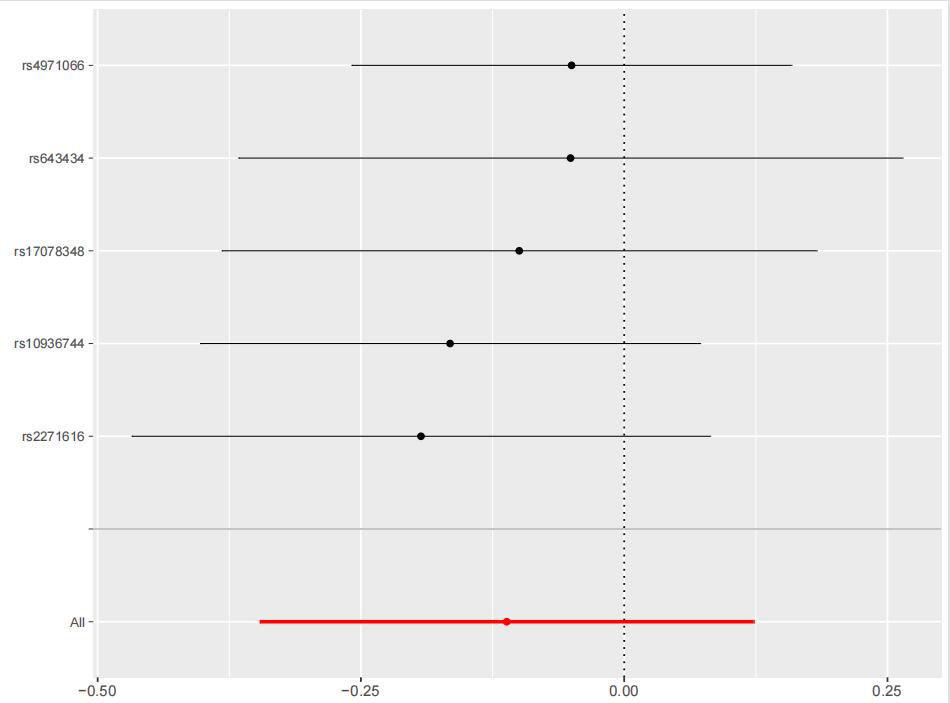


**Figure S3** Leave-one-out sensitivity analyses for COVID-19 susceptibility on bipolar disorder.


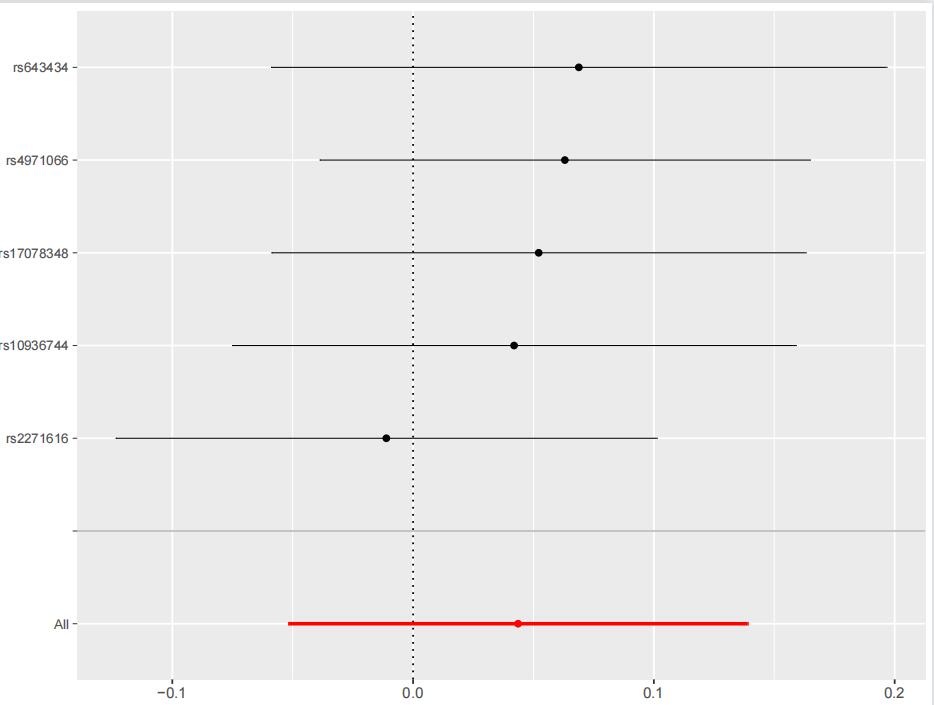


**Figure S4** Leave-one-out sensitivity analyses for COVID-19 susceptibility on major depressive disorder .


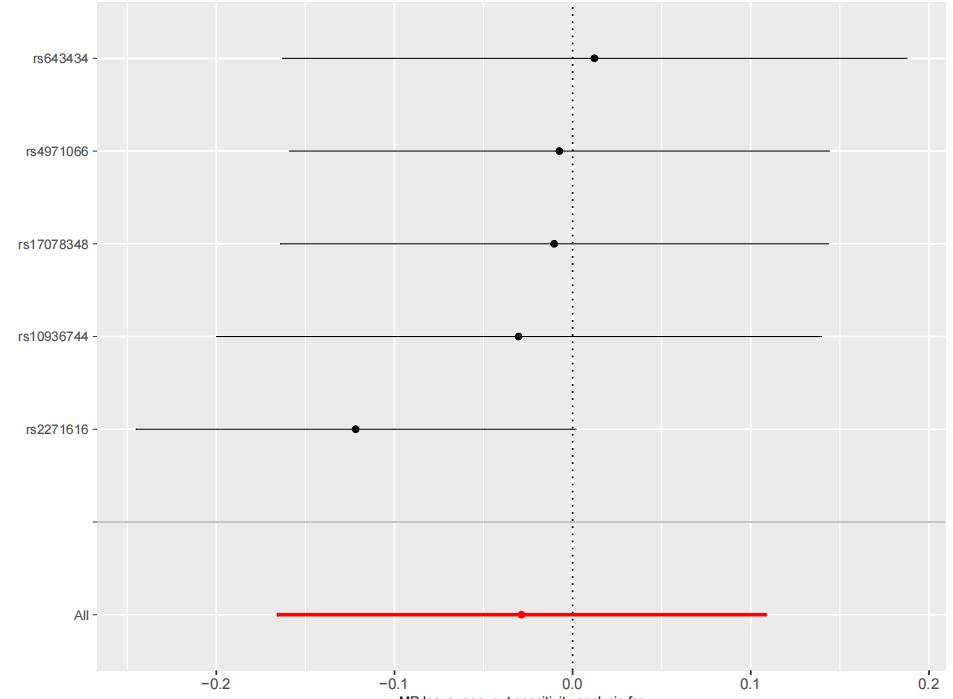


**Figure S5** Leave-one-out sensitivity analyses for COVID-19 susceptibility on schizophrenia.


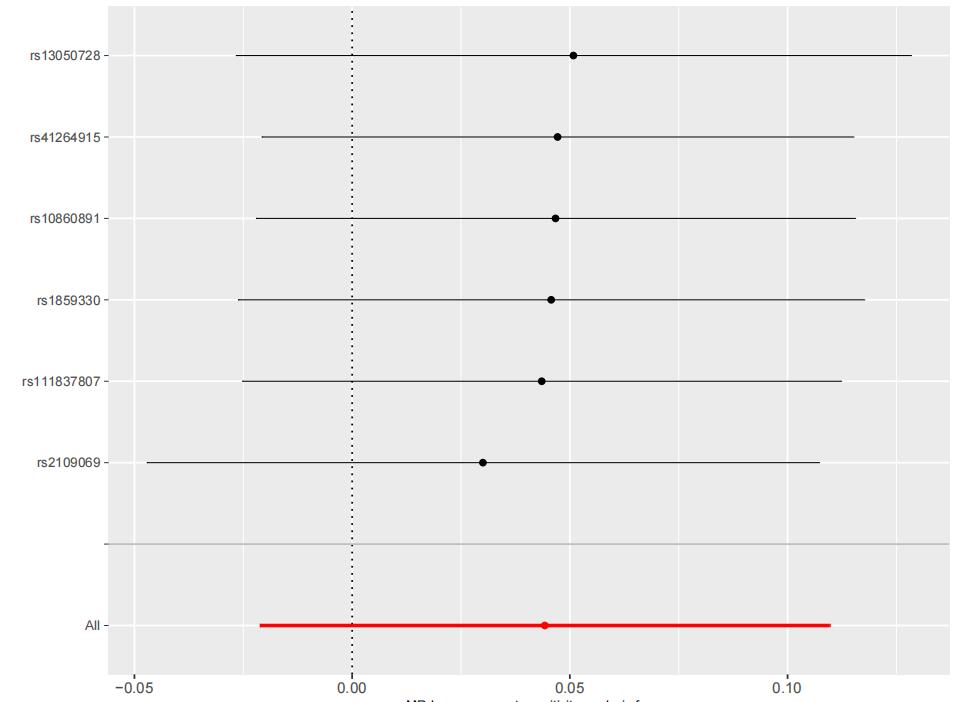


**Figure S6** Leave-one-out sensitivity analyses for COVID-19 hospitalization on anxiety disorders.


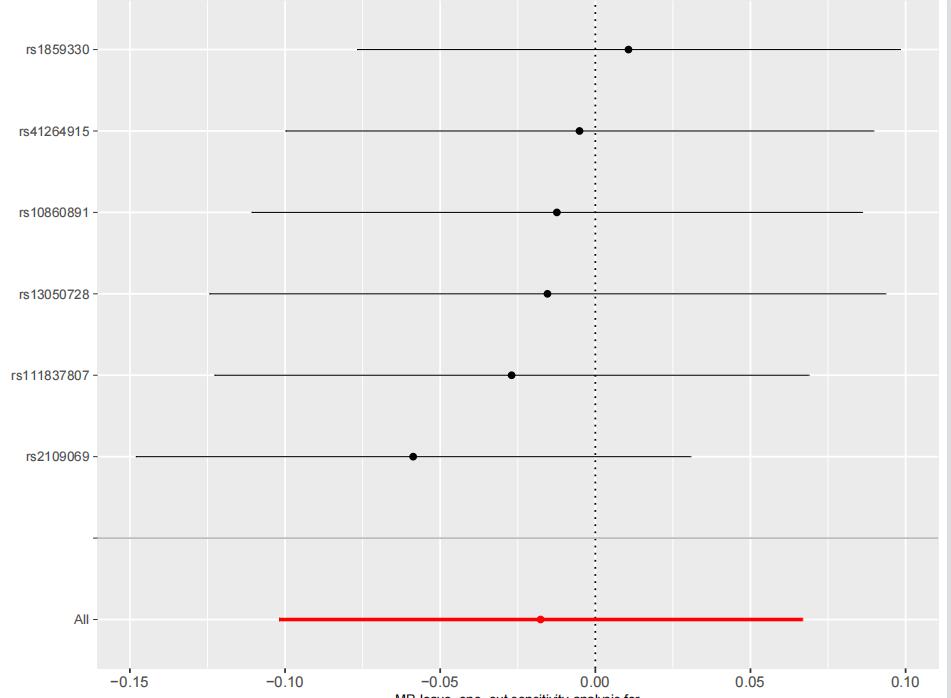


**Figure S7** Leave-one-out sensitivity analyses for COVID-19 hospitalization on autism spectrum disorder.


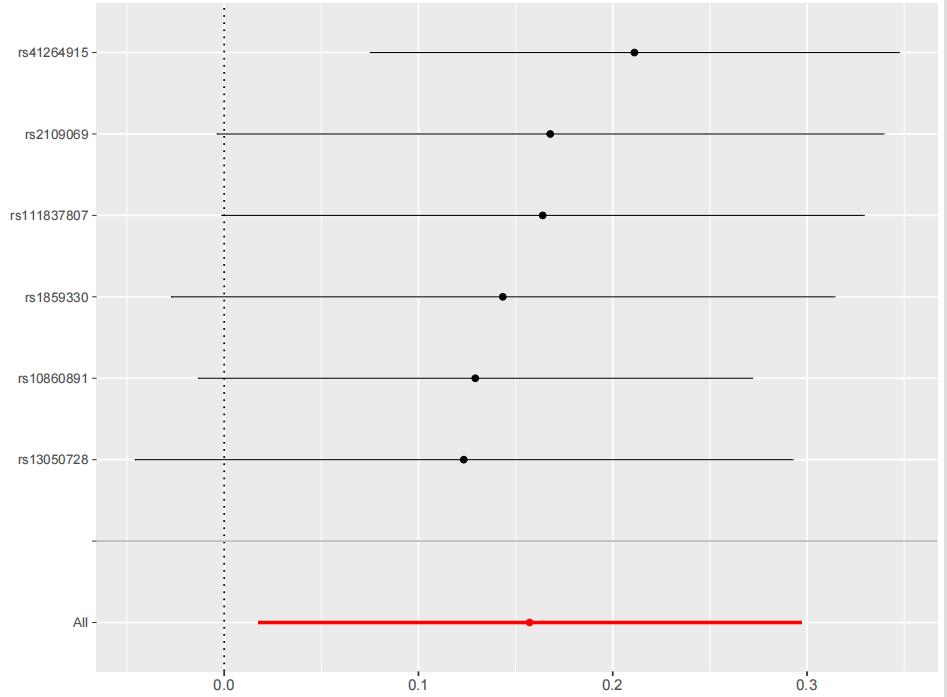


**Figure S8** Leave-one-out sensitivity analyses for COVID-19 hospitalization on bipolar disorder.


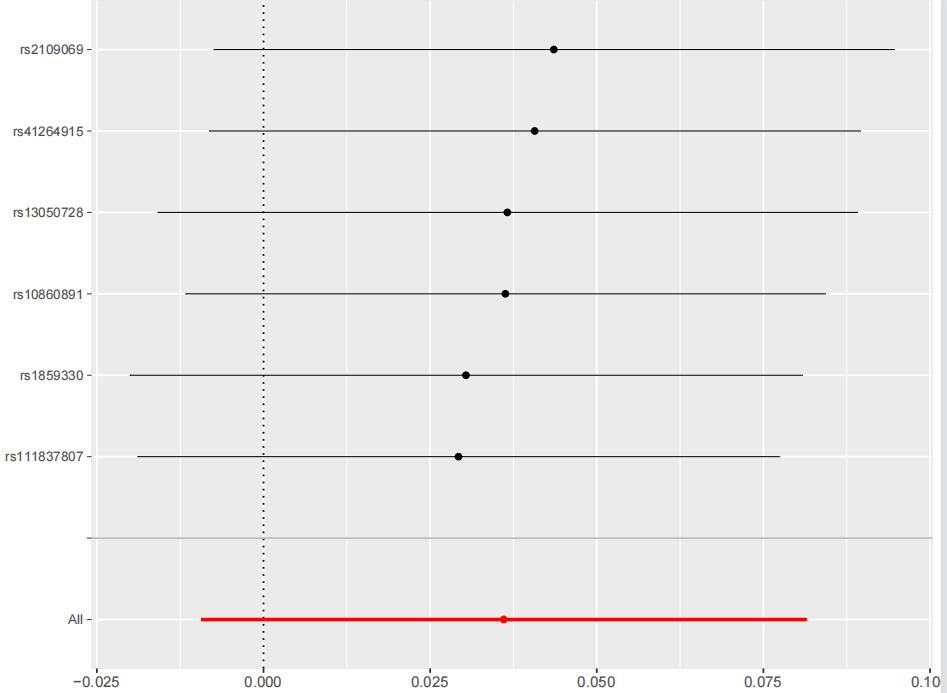


**Figure S9** Leave-one-out sensitivity analyses for COVID-19 hospitalization on major depressive disorder .


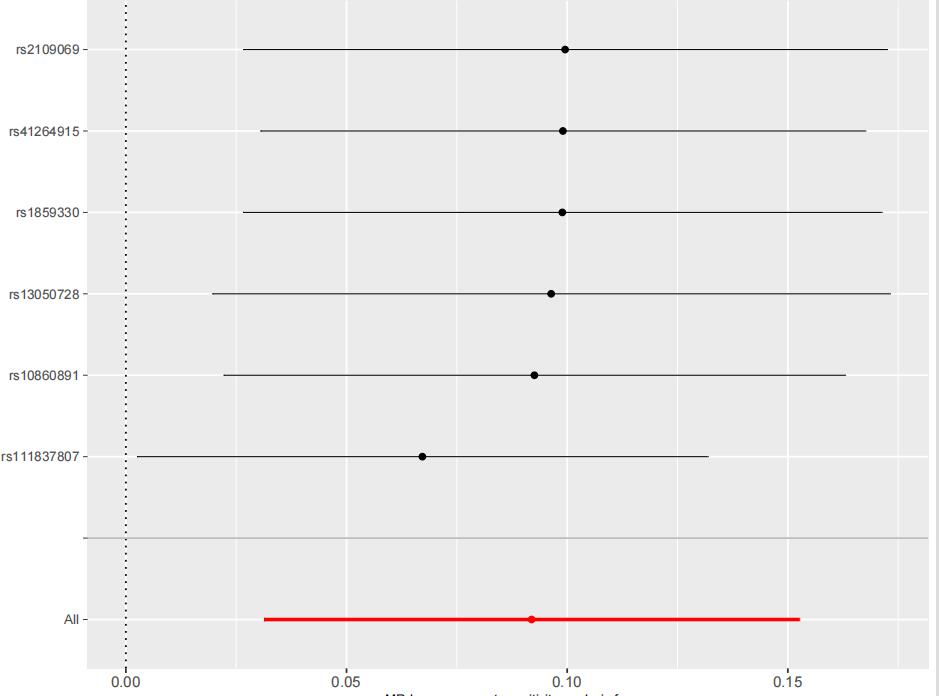


**Figure S10** Leave-one-out sensitivity analyses for COVID-19 hospitalization on schizophrenia.


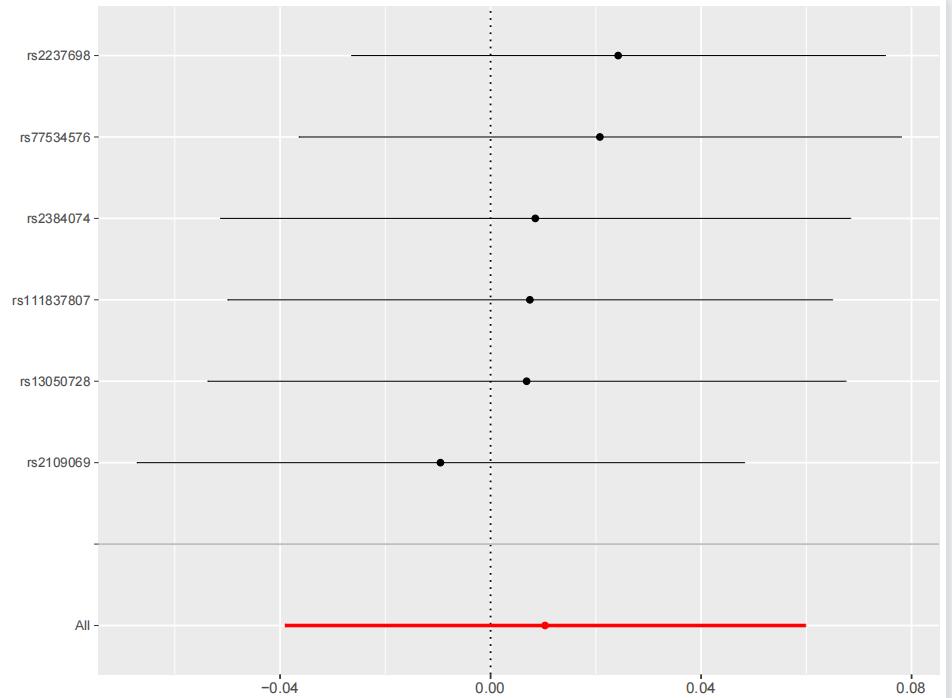


**Figure S11** Leave-one-out sensitivity analyses for COVID-19 severity on anxiety disorders.


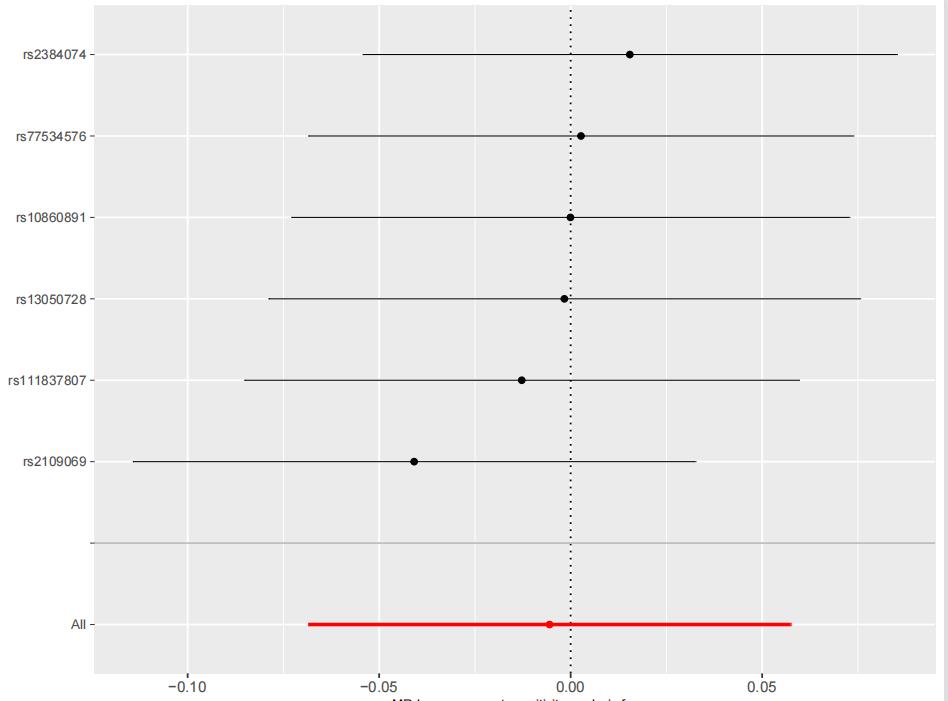


**Figure S12** Leave-one-out sensitivity analyses for COVID-19 severity on autism spectrum disorder.


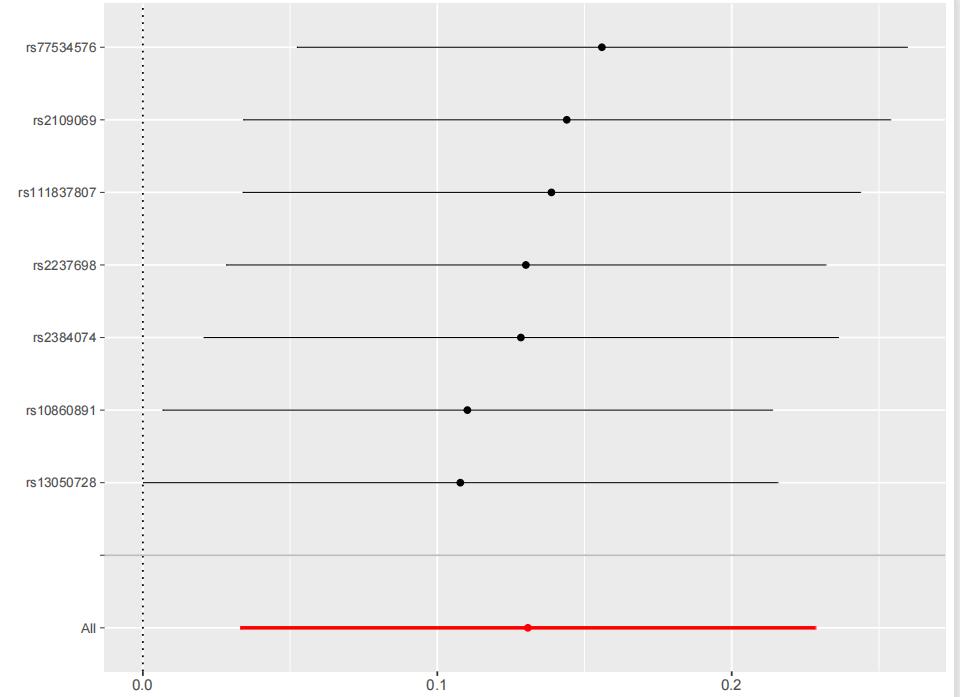


**Figure S13** Leave-one-out sensitivity analyses for COVID-19 severity on bipolar disorder.


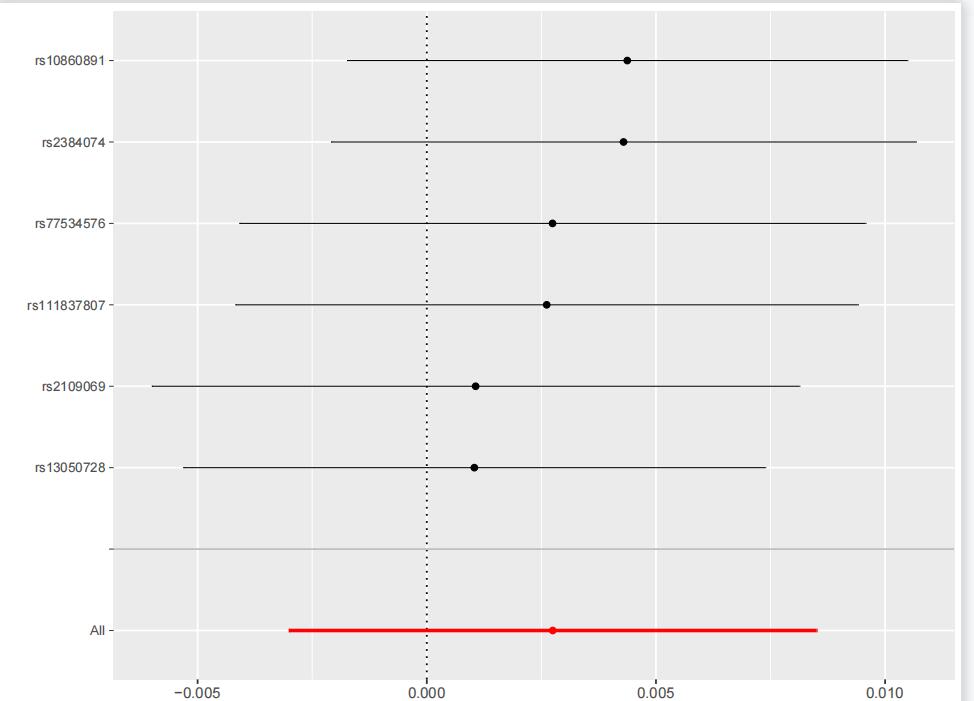


**Figure S14** Leave-one-out sensitivity analyses for COVID-19 severity on major depressive disorder .


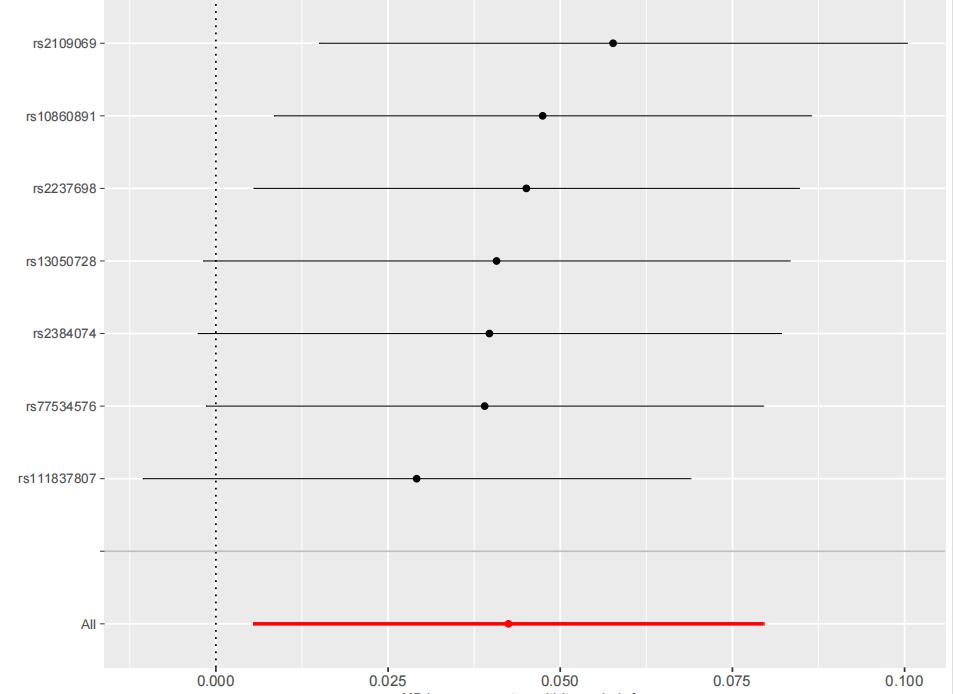


**Figure S15** Leave-one-out sensitivity analyses for COVID-19 severity on schizophrenia.
